# Computational principles of syntax in the regions specialized for language: integrating theoretical linguistics and functional neuroimaging

**DOI:** 10.3389/fnbeh.2013.00204

**Published:** 2013-12-18

**Authors:** Shinri Ohta, Naoki Fukui, Kuniyoshi L. Sakai

**Affiliations:** ^1^Department of Life Sciences, Graduate School of Arts and Sciences, The University of TokyoTokyo, Japan; ^2^Japan Society for the Promotion of ScienceTokyo, Japan; ^3^Department of Linguistics, Sophia UniversityTokyo, Japan; ^4^CREST, Japan Science and Technology AgencyTokyo, Japan; ^5^Department of Basic Science, Graduate School of Arts and Sciences, The University of TokyoTokyo, Japan

**Keywords:** syntax, universal grammar, recursive computation, inferior frontal gyrus, supramarginal gyrus, fMRI

## Abstract

The nature of computational principles of syntax remains to be elucidated. One promising approach to this problem would be to construct formal and abstract linguistic models that parametrically predict the activation modulations in the regions specialized for linguistic processes. In this article, we review recent advances in theoretical linguistics and functional neuroimaging in the following respects. First, we introduce the two fundamental linguistic operations: Merge (which combines two words or phrases to form a larger structure) and Search (which searches and establishes a syntactic relation of two words or phrases). We also illustrate certain universal properties of human language, and present hypotheses regarding how sentence structures are processed in the brain. Hypothesis I is that the Degree of Merger (DoM), i.e., the maximum depth of merged subtrees within a given domain, is a key computational concept to properly measure the complexity of tree structures. Hypothesis II is that the basic frame of the syntactic structure of a given linguistic expression is determined essentially by functional elements, which trigger Merge and Search. We then present our recent functional magnetic resonance imaging experiment, demonstrating that the DoM is indeed a key syntactic factor that accounts for syntax-selective activations in the left inferior frontal gyrus and supramarginal gyrus. Hypothesis III is that the DoM domain changes dynamically in accordance with iterative Merge applications, the Search distances, and/or task requirements. We confirm that the DoM accounts for activations in various sentence types. Hypothesis III successfully explains activation differences between object- and subject-relative clauses, as well as activations during explicit syntactic judgment tasks. A future research on the computational principles of syntax will further deepen our understanding of uniquely human mental faculties.

## Introduction

**Figure 1:**
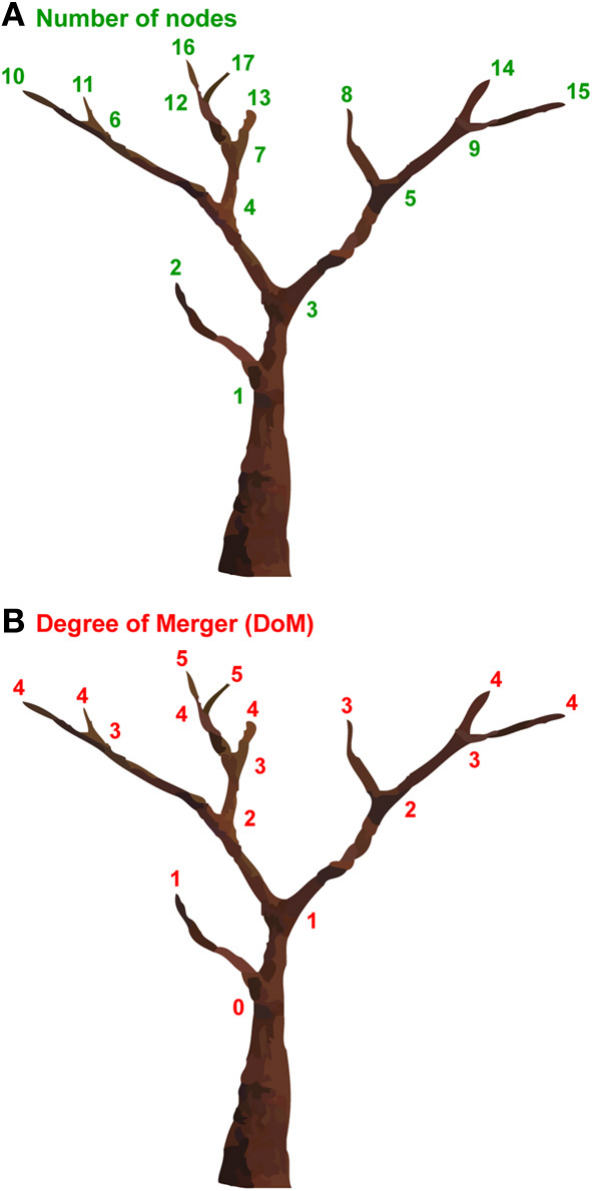
**Two models for measuring the complexity of tree structures. (A)** “The number of nodes” counts the total number of nonterminal nodes (branching points) and terminal nodes of a tree structure. The number of nodes of the tree structure shown is 17. **(B)** “The Degree of Merger (DoM)” quantifies the maximum depth of merged subtrees, or the degree of branching. We increased the number one by one for each node, starting from the trunk (zero) to terminal nodes. The DoM of the tree structure shown is 5.

Tree structures are one of the most ubiquitous structures in nature, appearing in the branchings of rivers, lightning, snowflakes, trees, blood vessels, nervous systems, etc., and can be simulated in part by fractal geometry (Mandelbrot, 1977). To properly quantify the complexity of such tree structures, various models have been proposed. The number of nodes would be one of the simplest models; this approach consists of simply counting the total number of non-terminal nodes (branching points) and terminal nodes of a tree structure (Figure 1A). This model obviously cannot capture hierarchical levels within the tree (sister relations in linguistic terms). To properly measure the hierarchical levels of a tree structure, we have proposed the Degree of Merger (DoM) as a key computational concept (Figure 1B) (Ohta et al., 2013). The DoM is defined as the *maximum depth* of merged subtrees (called Mergers) within a given domain. With this model, the same numbers are assigned to the nodes with an identical hierarchical level. The DoM corresponds to the number of iterations for generating fractal figures, when the tree structures are self-similar.

In this article, we first explain certain universal properties of human language discovered in modern linguistics, and we present hypotheses regarding how sentence structures are processed in the brain. We then introduce our recent functional magnetic resonance imaging (fMRI) study, which demonstrated that the DoM is indeed a key syntactic factor that accounts for syntax-selective activations in the regions specialized for language (Ohta et al., 2013). We also show that the top-down connectivity from the left inferior frontal gyrus to the left supramarginal gyrus is critical for the syntactic processing. Next, we clarify that the DoM can account for activation modulations in the frontal region, depending on different sentence structures. Finally, we hypothesize that the DoM domain changes dynamically in accordance with iterative Merge applications, the distance required for Search operations (or simply the “Search distance”), and/or task requirements. This hypothesis accounts for activation differences between subject-relative and object-relative clauses, as well as for activations during explicit syntactic judgment tasks.

## Universal properties of human language

### Theoretical background

Modern linguistics has clarified universal properties of human language, which, directly or indirectly, reflect the computational power, or engine, of the human language faculty. A sentence is not a mere string of words, but is made of phrase structure (called constituent structure). Moreover, a single phrase contains the key element (i.e., the “head”) that determines the basic properties of the phrase. Furthermore, a sentence can be recursively embedded within other sentences, as in, e.g., “*I think that John believes that Mary assumes that…*,” and there is in principle no upper bound for the length of sentences. These universal properties can be adequately and minimally expressed by hierarchical tree structures with a set of relevant structural relations defined on such structures (Chomsky, 1957, 1965).

To construct hierarchical tree structures, modern linguistics has proposed the fundamental linguistic operation of *Merge* (capitalized in linguistics to indicate a formal operation). Merge is a structure-building operation that combines two syntactic objects (words or phrases) to form a larger structure (Chomsky, 1995). Merge would be theoretically “costless,” requiring no driving force for its application (Saito and Fukui, 1998; Chomsky, 2004; Fukui, 2011). Besides Merge, we have proposed *Search* operation of searching syntactic features, which applies to a syntactic object already constructed by Merge, where Search couples and connects two distinct parts of the same structure, thereby assigning relevant features from one to the other part (Fukui and Sakai, 2003). Various other “miscellaneous” operations that have been employed in the linguistics literature, such as Agree, Scope determination, Copy, etc., are in fact different manifestations of one and the same, i.e., more generalized, operation of Search (Fukui and Sakai, 2003). Human language, therefore, should minimally contain two universal operations, Merge and Search. The total number of Merge and Search applications within an entire sentence are here simply denoted as “number of Merge” and “number of Search,” respectively. The number of Merge in a sentence becomes always one less than the number of terminal nodes, *irrespective of sentence structures* (see Appendix S2 of Ohta et al., 2013).

### Symbol sequences and formal languages

In regard to formal symbol sequences beyond the bounds of finite state languages, three specific types of language have been discussed in the linguistics literature: (i) “counter language,” (ii) “mirror-image language,” and (iii) “copying language” (cf. Chomsky, 1957, p. 21).

(i) *ab*, *aabb*, *aaabbb*, …, and in general, all sentences consisting of *n* occurrences of *a* followed by *n* occurrences of *b* and only these;
(ii) *aa*, *bb*, *abba*, *baab*, *aaaa*, *bbbb*, *aabbaa*, *abbbba*, …, and in general, all sentences consisting of a string *X* followed by the “mirror image” of *X* (i.e., *X* in reverse), and only these;
(iii) *aa*, *bb*, *abab*, *baba*, *aaaa*, *bbbb*, *aabaab*, *abbabb*, …, and in general, all sentences consisting of a string *X* of *a*'s and *b*'s followed by the identical string *X*, and only these.

The counter language can be handled by a counting mechanism to match the number of each symbol, whereas the mirror-image language contains a mirror-image dependency, requiring more than a mere counter. If the number of symbols is not fixed (i.e., infinite), both of these languages are beyond the bounds of finite-state grammars, and are to be generated by context-free (simple) phrase structure grammars, while the copying language with a cross-serial dependency clearly goes beyond the bounds of even context-free phrase structure grammars, requiring a more powerful device, viz., context-sensitive phrase structure grammars or transformational grammars (Chomsky, 1959; Hopcroft and Ullman, 1979).

It remains a central issue in cognitive sciences whether or not the faculty of language is also shared by animals. Animals have thus been tested with regular symbol sequences such as A^*n*^B^*n*^ (*n* ≥ 2; i.e., AABB, AAABBB, …) and (AB)^*n*^ (*n* ≥ 2; i.e., ABAB, ABABAB, …), which differ in *symbol order*. In an animal study, songbirds were trained to discriminate patterns of A^*n*^B^*n*^ and (AB)^*n*^ in more than ten thousand trials (Gentner et al., 2006). However, this learning can be achieved by tracking symbol repetition or counting strategy alone (Corballis, 2007). There is also a recent report that songbirds seemed to discriminate strings with or without nesting (Abe and Watanabe, 2011), but this learning can be achieved by simply remembering partial strings (Beckers et al., 2012). Along the lines of contrasting A^*n*^B^*n*^ and (AB)^*n*^, fMRI studies have tested participants with different symbol sequences, such as A_2_A_1_B_1_B_2_ vs. A_1_B_1_A_2_B_2_ (each subscript denotes a matching order), which also differ in matching order (Bahlmann et al., 2008). The difference in activation patterns can be simply explained by differences in any factor associated with matching orders and symbol orders, i.e., temporal order-related factors. It is thus necessary to completely control these general factors when extracting any syntactic factor from a number of cognitive factors involved in actual symbol processing.

Since the number of symbols is inevitably fixed (i.e., finite) in any actual experiment, it should be noted that any symbol sequence can be expressed by a regular (finite state) grammar, i.e., the least powerful grammar in the so-called Chomsky hierarchy. Therefore, one cannot, in principle, claim from the experiments that individual grammars (e.g., context-free phrase structure grammars vs. regular grammars) are differentially represented in the brain. Thus, the neural representation of individual grammars was *not* within the scope of Ohta et al. (2013). In addition to the various models examined, other non-structural and non-symbolic models with simple recurrent networks have been proposed to process some examples of even context-free and context-sensitive phrase structure languages, generalizing to some degree to longer strings than the training set (Rodriguez, 2001). However, these models do not account for any parametric modulation of the activations reported in Ohta et al. (2013), except the length of sentences.

**Figure 2:**
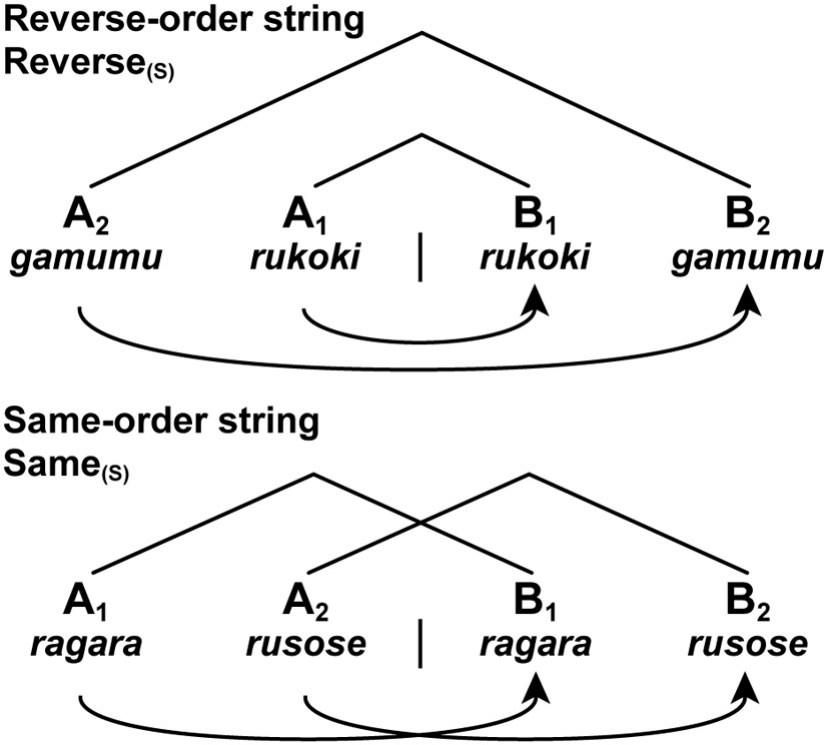
**Two basic types of letter strings related to formal languages.** We tested two string conditions with short [(S) as a subscript] stimuli: Reverse_(*S*)_ and Same_(*S*)_. Each letter string was formed by jumbling letters of either a pseudonoun or pseudoverb (see Figure 4). We also tested the long stimuli with six items. Each curved arrow with an arrowhead denotes a Search operation, as in the following figures. Symbols used: A, sample stimulus; B, comparison stimulus.

In the previous experiment, we introduced letter strings, which had no lexical associations but had both symbol orders (e.g., AABB and ABAB) and matching orders (e.g., A_2_A_1_B_1_B_2_). There were two basic types of strings: reverse-order strings (Reverse) and same-order strings (Same). In the Reverse strings, the first and second halves of a string were presented in the reverse order, while in the Same strings the halves were presented in the same order (Figure 2). Under these conditions, there was actually no path connecting the non-terminal nodes of symbol pairs (e.g., A_1_B_1_ and A_2_B_2_), as there was *no* Merge application to connect the multiple pairs. In regard to the symbol orders, both the Reverse and Same strings took the above type (i) of A^*n*^B^*n*^. In regard to the matching orders, the Reverse string took the type (ii) of A_2_A_1_B_1_B_2_ or A_3_A_2_A_1_B_1_B_2_B_3_, while the Same string took the type (iii) of A_1_A_2_B_1_B_2_ or A_1_A_2_A_3_B_1_B_2_B_3_.

### Hypothesis I

Given a tree structure with a formal property of Merge and *iterativity* (recursiveness) (Fukui, 2011), we propose the following hypothesis (Hypothesis I):

(1) The DoM, which can be defined as the *maximum depth* of merged subtrees within a given domain, is a key computational concept to properly measure the complexity of tree structures.

The DoM can quantify and compare various syntactic phenomena, such as self-embedding, scrambling, *wh*-movement, etc. Furthermore, when Search applies to each syntactic object with its hierarchical structure, the calculation of the DoM plays a critical role. Indeed, from a nested sentence “[[*The boy*_2_ [*we*_3_ *like*_3_]_2_]_1_ *sings*_1_]_0_” (subscripts denote the DoM for each node), two sentences “[*The boy.*..]_1_* sings*_1_” and “*we*_3_ *like*_3_” are obtained, where relevant features (numbers and persons here) are searched and matched between the nodes with the identical DoM. Since such analyses of hierarchical structures would produce specific loads in syntactic computation, we expect that the DoM and associated “number of Search” would affect performances and cortical activations.

Sentences with various constructions have been previously discussed in terms of the acceptability of sentences (cf. Chomsky, 1965, p. 12).

(i) nested constructions
(ii) self-embedded constructions
(iii) multiple-branching constructions
(iv) left-branching constructions
(v) right-branching constructions

The nested constructions are created by *centrally* embedding a phrase within another phrase (with some non-null element to its left and some non-null element to its right), and the self-embedded constructions are the special case of nested constructions when nesting occurs within the *same* type of phrases (e.g., noun phrases). The multiple-branching constructions are made by conjoining phrases at the same hierarchical level, and the left/right-branching constructions are yielded by merging a phrase in the left-most or right-most phrase. The degrees of nesting and self-embedding have already been proposed to model the understanding of sentences (Miller and Chomsky, 1963). By generalizing this attractive idea in such a way as to include any construction with merged phrases, we introduced the DoM as a key computational concept.

**Figure 3:**
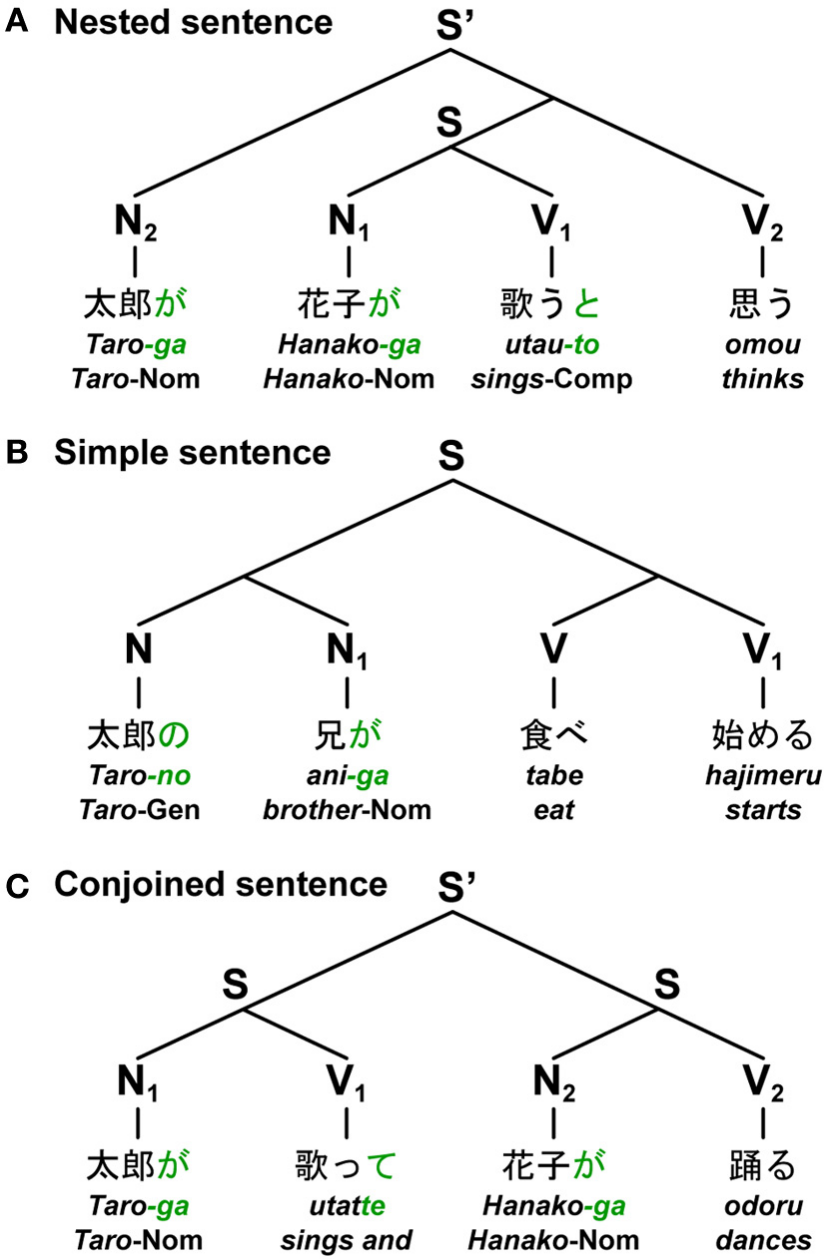
**Japanese sentences with three major constructions.** The figure shows three basic types of sentences in Japanese: the nested sentence, simple sentence, and conjoined sentence. Based on contemporary linguistics, each diagram represents a unique tree structure of each sentence constructed from nouns and verbs. Below each example, word-by-word translations in English are shown. **(A)** A sentence (S) at the lowest hierarchical level was nested into an entire sentence (S') (“*Taro-ga Hanako-ga utau-to omou*,” “*Taro thinks that Hanako sings*”). **(B)** A simple sentence was constructed by adding the same number of left/right branches to both nouns and verbs (“*Taro-no ani-ga tabe hajimeru*,” “*Taro's brother starts eating*”). **(C)** An entire sentence (S') was constructed by conjoining two sentences (“*Taro-ga utatte Hanako-ga odoru*,” “*Taro sings, and Hanako dances*”). Symbols used: S and S', sentence; N, noun phrase; V, verb phrase; -*ga*, nominative case marker; -*no*, genitive case marker; -*to*, complementizer; -*te*, gerundive form; Nom, nominative case; Gen, genitive case; Comp, complementizer.

Based on the nested (self-embedded), left/right-branching, and multiple-branching constructions, three basic types of sentences can be distinguished: the nested sentence (Nested), simple sentence (Simple), and conjoined sentence (Conjoined), respectively. The sentences shown in Figure 3 are some examples in Japanese. Given syntactic structures like the ones shown, the correspondence of each subject-verb pair becomes fixed. Here N and V denote a noun phrase and a verb phrase, respectively. For the sentence shown in Figure 3A, an entire sentence is constructed by nesting sentences in the form of [N_2_[N_1_ V_1_]V_2_], where [N_*i*_ V_*i*_] represents a subject-verb pair of a sentence. Since Japanese is a head-last, and hence an SOV (verb-final) language, a main verb is placed after a subordinate clause. Therefore, Japanese sentences naturally yield nested structures without having to employ, as in English, object-relative clauses (e.g., “*The boy who_i_ we like t_i_ sings*”), which require “movement” of an object (i.e., with more Merge applications) and thus leave behind a “trace” (*t_i_*, subscripts denote the same entity). For the sentence shown in Figure 3B, a simple sentence is constructed by adding the same number of left/right branches to both Ns and Vs. The last noun (i.e., head) in the branches of Ns made a subject-verb pair with the last verb (i.e., head) of a compound verb. Each simple sentence thus takes the form of [(NN_1_)(VV_1_)]. For the sentence shown in Figure 3C, an entire sentence is constructed by conjoining sentences in the form of [N_1_V_1_][N_2_V_2_]. When considering longer sentences like N_3_N_2_N_1_V_1_V_2_V_3_, these constructions have distinct values for DoM.

### Hypothesis II

In any sentence, functional elements, such as inflections, auxiliary verbs, and grammatical particles, serve an essentially grammatical function without descriptive content. In regard to the fundamental role of these functional elements, we propose the following hypothesis (Hypothesis II):

(2) The basic frame of the syntactic structure of a given linguistic expression (e.g., sentence) is determined essentially by functional elements, which trigger Merge and Search operations.

In the non-sense poem “Jabberwocky” by Lewis Carroll, e.g., “'*Twas* (’*It was*')* brillig, and the slithy toves did …*,” the basic frames of syntactic structures are indeed determined by the functional elements of “*'Twas*,” “*and*,” “*the*,” “-*s*,” and “*did*.” In the Japanese language, grammatical particles and morphosyntactic inflections are functional elements. The sentences shown in Figure 3 actually contain only three kinds of grammatical particles, which represent *canonical* (i.e., in a prototypical use) case markings and syntactic information in Japanese: -*ga*, a nominative case marker; -*no*, a genitive case marker; and -*to*, a complementizer. It should be noted that both the nested and simple sentences have the same symbol order of N^*n*^V^*n*^, but they have different grammatical particles and syntactic structures. In contrast, both the simple and conjoined sentences have the same tree structures as a result, but they have different symbol orders of N^*n*^V^*n*^ or (NV)^*n*^ (*n* ≥ 2). It is the grammatical particles and morphosyntactic inflections, but not symbol orders or matching orders themselves, that determine the basic frame of syntactic structures of a sentence.

**Figure 4:**
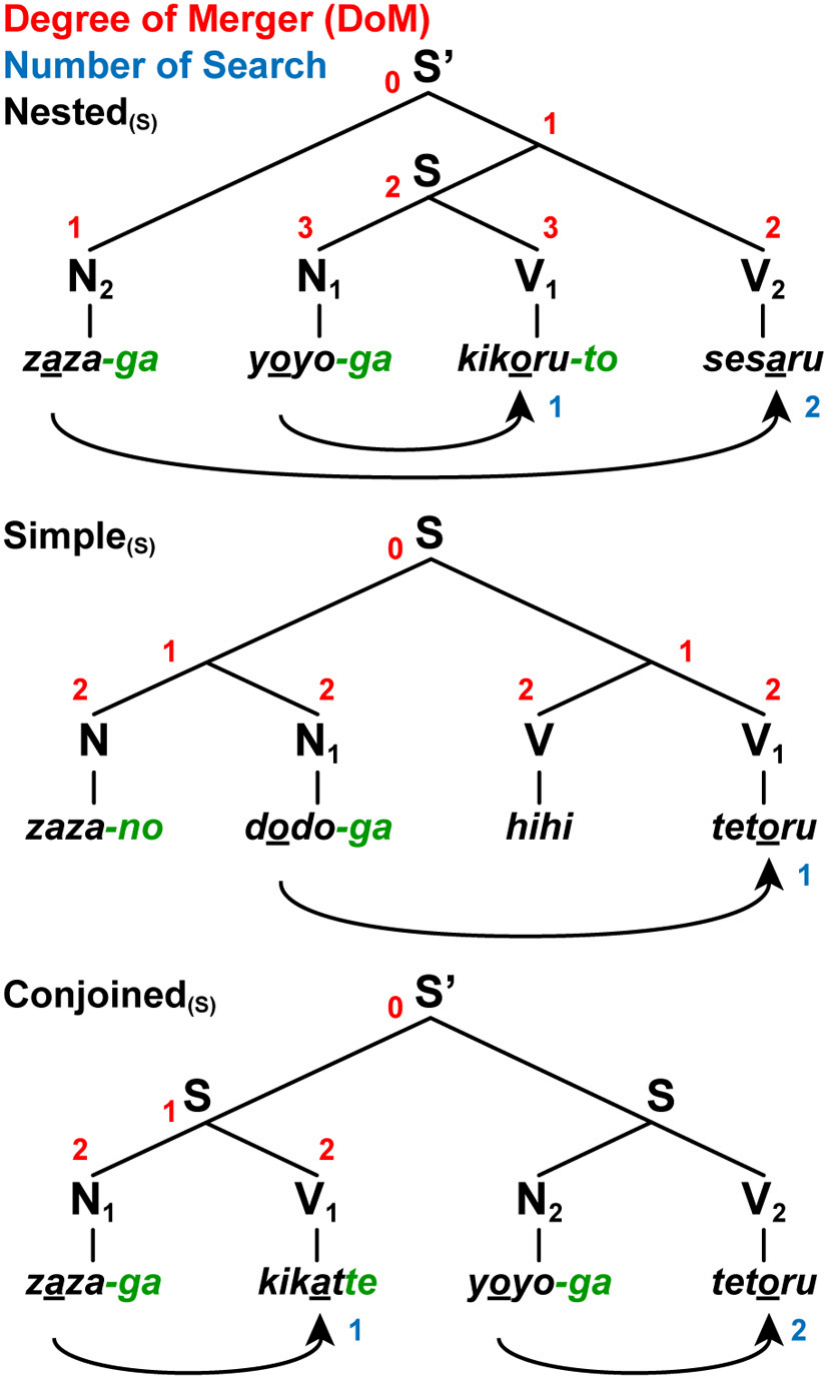
**A paradigm for testing various sentence structures.** We tested three sentence conditions with short [(S) as a subscript] jabberwocky sentences: Nested_(*S*)_, Simple_(*S*)_, and Conjoined_(*S*)_. Note the syntactic structures of these jabberwocky sentences are same as those of real sentences in Figure 3. The digits shown in red and blue denote the DoM for each node and “number of Search,” respectively. We also tested the long stimuli with six words.

Following morphosyntactic and phonological features of Japanese verbs (Tsujimura, 2007), Vs take a non-past-tense form (-*ru*), past-tense form (-*ta*), or gerundive form (-*te*); Vs ending with -*to* and -*te* introduce *that*-clauses and *and*-conjunctives, respectively. The gerundive form can be used not only in *and*-conjunctives, but in compound verbs (e.g., “*tabete-simau*,” “*finish eating*”; actual Japanese words will be translated hereafter), much as gerunds can in English. The -*ga*, -*no*, -*to*, and -*te* endings (*green* letters in Figures 3, 4), together with the first verb of a compound verb in an adverbial form (e.g., “*tabe*”), are associated with Merge applications to connect multiple nouns/verbs or sentences, amounting to “number of Merge.” The Japanese language lacks the “agreement features” (i.e., number, person, gender, etc.), but it is nevertheless equipped with the general Search procedure that is employed in agreement phenomena in other languages. This Search mechanism is in fact attested for various other phenomena in Japanese (see Fukui and Sakai, 2003 for further discussion). For example, the Japanese language exhibits a phenomenon called “honorification,” where a noun phrase denoting an honored person and the form of honorifics in verbs are to be matched (Gunji, 1987; Ivana and Sakai, 2007).

In this section, we provided some theoretical discussions based on modern linguistics, focusing on the two fundamental linguistic operations of Merge and Search. We hypothesized that the DoM is a key computational concept to properly quantify the complexity of tree structures, and that the basic frame of the syntactic structure of a given linguistic expression is determined essentially by grammatical particles and morphosyntactic inflections, which trigger Merge and Search operations.

## The DoM as a key syntactic factor elucidated by an fMRI experiment

One possible way to elucidate the neural basis of computational properties of natural language is to examine how the brain responds to the modulation of specified syntactic factors. We should not be content with such a general cognitive factor as so-called “syntactic complexity” or “syntactic working memory,” which could involve both linguistic and non-linguistic factors. We should instead identify minimal factors that *sufficiently* explain any activation change obtained. In our recent study, we focused on different sentence constructions, and found that the DoM and “number of Search” were the *minimal* syntactic factors associated with phrase structures, which parametrically modulate cortical responses measured with event-related fMRI (Ohta et al., 2013). In this section, we will present the basic paradigm and results of this work.

### A paradigm to test hypotheses I and II

We used jabberwocky sentences, which consist of pseudonoun phrases (Ns) and pseudoverb phrases (Vs) that lack lexical associations, but have grammatical particles and morphosyntactic inflections (Figure 4). According to Hypothesis II stated above, these jabberwocky sentences had the same syntactic structures as normal sentences. Under the sentence conditions of Nested, Simple, and Conjoined with the same structures shown in Figure 3, the jabberwocky sentences were visually presented in a phrase-by-phrase manner to the participants. We made six pseudonouns by repeating the same syllables with voiced consonants and any one of /a/, /u/, or /o/: *rara*, *zaza*, *mumu*, *gugu*, *yoyo*, and *dodo*. We also made four pseudoverb roots by repeating the same syllables with voiceless consonants and either /i/ or /e/: *kiki*, *hihi*, *sese*, and *tete*. Here, vowel harmony was adopted to change the last, i.e., the second, vowel of the verb root, so that this vowel harmonized with the vowel (i.e., /a/, /u/, or /o/) of the corresponding subject (e.g., “*rara-ga tetaru*” from “*teteru*,” underlined vowels within pseudowords). These features of vowels were only *experimentally* introduced, and these pseudoverbs lacked grammatical features, as in the Japanese verbs. In all jabberwocky sentences, the distinction between Ns and Vs was clear without memorizing pseudowords, because Ns, but not Vs, ended with either -*ga* or -*no*, i.e., case markers in Japanese such as -*ga* and -*no* can be generally attached only to nominal phrases.

To test whether participants actually paid attention to the correspondence of each subject-verb pair, we used a matching task, such that the vowel of a subject (N_*i*_ as a sample stimulus) was matched with the last vowel of the corresponding verb root (V_*i*_ as a comparison stimulus), probing the goal with the same vowel as explained above. It follows that the same syntactic structures were constructed from matching stimuli and non-matching stimuli (e.g., “*rara-ga teturu*”), which were both well-formed, i.e., *grammatical*, in Japanese. A matching strategy (counting, for example, the first and the fourth stimuli for matching) was useful in solving the task, but performing the task was *not* prerequisite for constructing syntactic structures. Our matching task is different from classification tasks for symbol orders (e.g., AABB vs. ABAB, where A and B are symbols representing certain sets of stimuli), which can be solved by counting the maximum number of consecutively repeated symbols. The order of the Nested, Simple, and Conjoined was pseudo-randomized without repetition. We further examined whether cortical activations were modulated by the length of sentences: short (S as a subscript, e.g., Conjoined_(*S*)_; four-word) and long (L as a subscript, e.g., Conjoined_(*L*)_; six-word) sentences, where the DoM domain spanned four and six relevant words, respectively.

We also used the same matching task under the string conditions of Reverse and Same (Figure 2), such that the first half of a string (A_*i*_ as a sample stimulus) was matched with the corresponding second half (B_*i*_ as a comparison stimulus) in the reverse or same order. These string conditions also controlled any involvement of the matching strategy stated above. Between the Nested (N_2_N_1_V_1_V_2_) and Reverse (A_2_A_1_B_1_B_2_) conditions, the curved arrows shown in Figures 2, 4 represent the *same* matching order of sequentially presented stimuli. The symbol order was also identical among the Nested, Simple, Reverse, and Same conditions in the form of N^*n*^V^*n*^ or A^*n*^B^*n*^. Combining these multiple conditions, we were able to properly examine whether different structures were actually constructed between sentences and strings. The spatial and temporal resolution of fMRI, as well as its sensitivity, has been proven to be high enough to confirm various hypotheses about human cognitive functions like ours.

### Syntax-selective activations modulated by the DoM and the number of search

**Figure 5:**
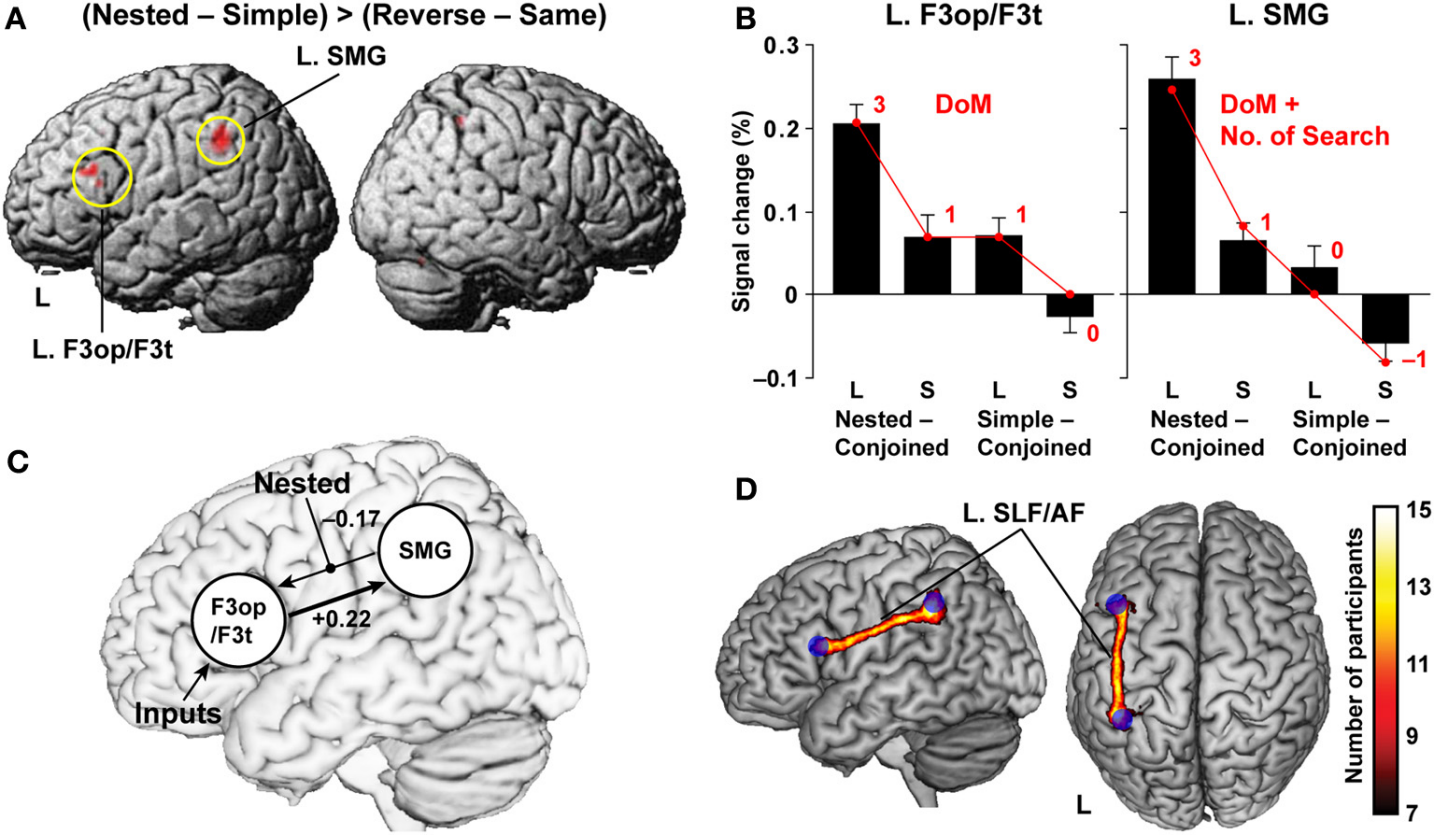
**Functional and anatomical evidence of a syntax-related network. (A)** Regions identified by the (Nested − Simple) > (Reverse − Same) contrast (see Figure 4). Activations were projected onto the left (L) and right lateral surfaces of a standard brain. **(B)** Percent signal changes for Nested − Conjoined and Simple − Conjoined in the L. F3op/F3t and L. SMG. Overlaid red dots and lines denote the values fitted with the estimates (digits in red) for the best models: DoM for the L. F3op/F3t and “DoM + number of Search” for the L. SMG. **(C)** The results of DCM, testing effective connectivity between the L. F3op/F3t and L. SMG. The best model included a significant top-down connection from the L. F3op/F3t to L. SMG (a thick line). **(D)** Anatomical connectivity between the L. F3op/F3t and L. SMG revealed by DTI. The population probability map is shown on the left lateral and dorsal surfaces of a standard brain with maximum intensity projection. Blue spheres represent seed regions of the L. F3op/F3t and L. SMG. Symbols used: L, long sentences; S, short sentences.

To control both matching orders and symbol orders, we directly compared the Nested with the Reverse condition, using the Simple and Same conditions as respective references, i.e., (Nested − Simple) > (Reverse − Same), where we combined the short and long stimuli. This contrast further controlled various linguistic and non-linguistic factors, such as the number of Merge, number of case markers, number of nodes, memory span, and counting. This point is particularly important, because temporal order-related or memory-related factors have often been confused with differences in structure or grammar type. Significant activation was elicited by this contrast in the pars opercularis and pars triangularis of the left inferior frontal gyrus (L. F3op/F3t) [local maximum: (*x*, *y*, *z*) = (−51, 24, 24), *Z* = 5.8], and the left supramarginal gyrus (L. SMG) [(−39, −45, 42), *Z* = 5.7] (Figure 5A). Our results are best explained by the linguistic factors associated with the Nested condition, supporting our second hypothesis that basic syntactic structures are constructed when well-formed sentences are given even without lexical meanings.

For these two critical regions, we examined the percent signal changes under the Nested and Simple conditions by subtracting those under the Conjoined condition, which had the simplest tree structures (Figure 4 and Table 1), separately for long and short sentences. Since we used the Conjoined_(*L*)_ and Conjoined_(*S*)_ as appropriate references, we examined whether likewise *subtracted* estimates of each factor (e.g., DoM for Nested_(*L*)_ – Conjoined_(*L*)_; see Table 1) directly explained the parametric modulation of activations in the four contrasts of Nested_(*L*)_ – Conjoined_(*L*)_, Nested_(*S*)_ – Conjoined_(*S*)_, Simple_(*L*)_ – Conjoined_(*L*)_, and Simple_(*S*)_ – Conjoined_(*S*)_. The percent signal changes in the L. F3op/F3t and L. SMG, averaged across significant voxels, indeed correlated exactly in a step-wise manner with the parametric models of the DoM [3, 1, 1, 0] and “DoM + number of Search” [3, 1, 0, −1], respectively (Figure 5B). By generalizing the role of Search, we assumed that Search applied to a subject-verb pair, where the relevant features (vowels here) are experimentally “inserted” (Ohta et al., 2013).

**Table 1:** **Estimates of various factors to account for activations in Ohta et al. (2013)**.

**Factor**	**Nested_*(L)*_**	**Nested_*(S)*_**	**Simple_*(L)*_**	**Simple_*(S)*_**	**Conjoined_*(L)*_**	**Conjoined_*(S)*_**
Degree of Merger (DoM)	5	3	3	2	2	2
No. of Search	3	2	2	1	3	2
No. of nodes	11	7	11	7	10	7
	**Nested_*(L)*_ − Conjoined_*(L)*_**	**Nested_*(S)*_ − Conjoined_*(S)*_**	**Simple_*(L)*_ − Conjoined_*(L)*_**	**Simple_*(S)*_ − Conjoined_*(S)*_**
DoM	3	1	1	0
DoM + No. of Search	3	1	0	−1
No. of Search	0	0	−1	−1
No. of nodes	1	0	1	0

Estimates under the Conjoined condition were subtracted from those under the other Nested and Simple conditions [e.g., DoM for Nested_(*L*)_ − Conjoined_(*L*)_, 5 − 2 = 3], separately for long and short sentences. We regarded “DoM + number of Search” (i.e., adding the estimates of two factors) as an additional factor.

We further examined 19 models proposed in theoretical linguistics, psycholinguistics, and natural language processing to verify that the models of the DoM and “DoM + number of Search” best explained the cortical activations (Ohta et al., 2013). All contrasts of Nested_(*L*)_ – Conjoined_(*L*)_, etc. predicted that the activations should be exactly zero when a factor produced no effect or load relative to the Conjoined. We thus adopted a no-intercept model, in which percent signal changes of each region were fitted with a single (thus minimal) scale parameter to a model of each factor using its subtracted estimates. For the four contrasts, a least-squares method was used to minimize the residual sum of squares (RSS) for the four fitted values (i.e., four estimates multiplied by a fitting scale) against the corresponding signal changes averaged across participants (Table 2).

**Table 2:** **Fittings and likelihood of various models tested in Ohta et al. (2013)**.

**Factor**	**RSS**	***r*^2^**	***P*-values for four contrasts**	**Log-likelihood**	**Likelihood ratio**
**L. F3op/F3t**					
*DoM	0.0007	0.99	0.17, 0.92, 0.97, 0.99	65.0	1.0
DoM + No. of Search	0.0065	0.88	0.0035, 0.064, 0.63, 0.88	59.2	3.1 × 10^−3^
No. of Search	0.052	<0.1	<0.0001, 0.018, 0.019, 0.031	33.4	2.0 × 10^−14^
No. of nodes	0.015	0.72	0.0050, 0.0082, 0.018, 0.17	53.7	1.2 × 10^−5^
**L. SMG**					
DoM	0.0063	0.92	0.013, 0.083, 0.44, 0.49	58.8	0.079
*DoM + No. of Search	0.0020	0.97	0.22, 0.30, 0.42, 0.62	61.4	1.0
No. of Search	0.075	<0.1	<0.0001, 0.0061, 0.045, 0.090	23.6	3.8 × 10^−17^
No. of nodes	0.033	0.56	0.0004, 0.0005, 0.0061, 0.013	40.1	6.0 × 10^−10^

Percent signal changes in the L. F3op/F3t and L. SMG were fitted with a single scale parameter to a model of each factor using its subtracted estimates (Table 1) for the four contrasts of Nested_(L)_ − Conjoined_(L)_, Nested_(S)_ − Conjoined_(S)_, Simple_(L)_ − Conjoined_(L)_, and Simple_(S)_ − Conjoined_(S)_. The P-values for the t-tests are shown in ascending order. The models with an asterisk resulted in the best fit of 19 models tested (four models are shown here) for explaining activations in the L. F3op/F3t or L. SMG, i.e., with the least residual sum of squares (RSS), largest coefficient of determination (r^2^), and larger P-values. The likelihood ratio was taken as the ratio of each model’s likelihood to the best model’s likelihood. The best models were by far more likely than the other models.

The model of the DoM for the L. F3op/F3t, as well as that of “DoM + number of Search” for the L. SMG, indeed produced by far the least RSS (≤0.0020) and largest coefficient of determination (*r*^2^) (≥ 0.97). Goodness of fit was further evaluated for each model by using a one-sample *t*-test (significance level at α = 0.0125, Bonferroni corrected) between the fitted value for each contrast and individual activations. The model of the DoM for the L. F3op/F3t, as well as that of “DoM + number of Search” for the L. SMG, produced no significant deviation for the four contrasts (*P* ≥ 0.17). To further take account of interindividual variability, we fitted “linear mixed-effects models” with individual activations, and found that the models of the DoM and “DoM + number of Search” were by far more likely for the L. F3op/F3t and L. SMG, respectively. Even if we took the Simple condition as a reference for subtracted estimates, we obtained the same results of best models. These results directly support Hypotheses I and II, such that the basic frame of syntactic structures are determined essentially by functional elements, whereas the DoM, together with the number of Search, is a key factor to properly quantify the complexity of the syntactic structures.

### The significance of the connectivity between the l. F3Op /F3t and l. SMG

It has been reported that the L. F3op/F3t is specialized for syntactic processing (Stromswold et al., 1996; Dapretto and Bookheimer, 1999; Embick et al., 2000; Hashimoto and Sakai, 2002; Friederici et al., 2003; Musso et al., 2003; Suzuki and Sakai, 2003; Kinno et al., 2008), suggesting that this region subserves a grammar center (Sakai, 2005). On the other hand, the left angular gyrus and SMG (L. AG/SMG) have been suggested to be important for vocabulary knowledge or lexical processing (Lee et al., 2007; Pattamadilok et al., 2010). To elucidate the relationships between the L. F3op/F3t and L. SMG, we modeled the effective connectivity between these two regions by using dynamic causal modeling (DCM). Our interest was to identify the direction of the connectivity modulated by the Nested condition, which has the largest DoM of all conditions. First, we assumed intrinsic, i.e., task-independent, bi-directional connections, and the models were grouped into three “modulatory families”: families with modulation for the bottom-up connection from the L. SMG to L. F3op/F3t, for the top-down connection from the L. F3op/F3t to L. SMG, and for both connections. Each family was composed of three “input models” as regards the regions receiving driving inputs. We found that the model with the modulation for the bottom-up connection, in which the L. F3op/F3t received driving inputs, was the best and most probable model (Figure 5C). We further confirmed that the intrinsic top-down connectivity was significantly positive (+0.22; *P* < 0.0002), while the bottom-up connectivity was negatively modulated.

A recent DCM study with a picture-sentence matching task has suggested that the L. F3op/F3t received driving inputs (den Ouden et al., 2012), which was consistent with our DCM results. Moreover, our previous studies revealed that the functional connectivity between the L. F3t/F3O (pars orbitalis) and L. AG/SMG was selectively enhanced during sentence processing (Homae et al., 2003), and that the L. AG/SMG was also activated during the identification of correct past-tense forms of verbs, probably reflecting an integration of syntactic and vocabulary knowledge (Tatsuno and Sakai, 2005). Considering the role of the L. AG/SMG in lexical processing, the Search operation based on the DoM would be essential in assigning relevant features to the syntactic objects derived from lexical items.

To further confirm the anatomical plausibility of the network between the L. F3op/F3t and L. SMG revealed by DCM, we used diffusion tensor imaging (DTI) with a probabilistic tractography. We observed that a single continuous cluster of the left superior longitudinal and arcuate fasciculi (SLF/AF) connected these regions (cluster size, 3189 mm^3^), together with much smaller clusters or islands (Figure 5D). Moreover, the left SLF/AF was consistently observed in all participants.

The findings of recent DTI studies have been controversial regarding the functional roles of two different pathways in language processes: the dorsal tracts of the SLF/AF, and the ventral tracts of the middle longitudinal fasciculus (MdLF) and extreme capsule (EmC). Both pathways connect the inferior frontal and superior/middle temporal areas (Saur et al., 2008; Wilson et al., 2011; Wong et al., 2011; Griffiths et al., 2013). Our DCM and DTI results indicate that the L. SMG activations reflecting the DoM mirrored a top-down influence from the L. F3op/F3t through the left dorsal pathway of the SLF/AF, revealing the most crucial network and pathway for syntactic computation.

## Further confirmation of hypotheses I and II

### A picture-sentence matching paradigm

**Figure 6:**
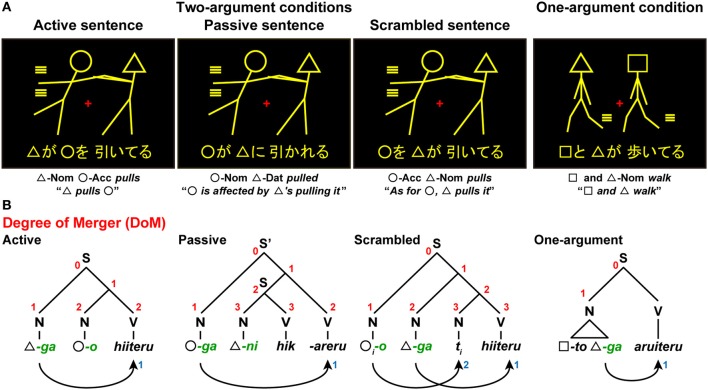
**A picture-sentence matching paradigm in Kinno et al. (2008). (A)** A picture-sentence matching task under either Two-argument conditions or a One-argument condition. Each stimulus consisted of one picture (top) and one sentence (bottom). Below each example, word-by-word and full translations in English are shown. An identical picture set was used under the Two-argument conditions, where we tested three sentence types: active sentences (“*∆-ga ◦-o hiiteru*”), passive sentences (“*◦-ga ∆-ni hik-areru*”), and scrambled sentences (“*°-o ∆-ga hiiteru*”). Under the One-argument condition, we presented syntactically simpler active sentences (“*□-to ∆-ga aruiteru*”). **(B)** The syntactic structures of three sentence types. The digits shown in red and blue denote the DoM for each node and “number of Search,” respectively. Symbols used: S and S', sentence; N, noun phrase; V, verb phrase; Nom, nominative case; Acc, accusative case; Dat, dative case; -*ga*, nominative case marker; -*o*, accusative case marker; -*ni*, dative case marker; -*to*, coordinator; *t_i_*, trace (subscripts denote the same entity).

We further examined whether our hypotheses hold for various cases discussed in previous studies. In our fMRI study (Kinno et al., 2008), we used a picture-sentence matching task with three sentence types in Japanese: active, passive, and scrambled sentences (Figure 6A). In the picture-sentence matching task, the participants read a sentence covertly and judged whether or not the action depicted in a picture matched the meaning of the sentence. Each sentence had two noun phrases called *arguments*, each of which assumes a different grammatical relation (“subject, direct object, or indirect object” in linguistic terms) and a semantic role (“agent, experiencer, or patient” in linguistic terms, i.e., an agent who performs the action, and an experiencer/patient who is affected by it); these three conditions were thus called Two-argument conditions. More specifically, the active, passive, and scrambled sentences corresponded to “agent and patient” (subject and direct object), “experiencer and agent” (subject and indirect object), and “patient and agent” (direct object and subject) types, respectively. Pictures consisted of two stick figures, each of which was distinguished by a “head” symbol: a circle (◦), square (□), or triangle (∆). These sentences excluded the involvement of pragmatic information about word use (e.g., “*An officer chases a thief*” is more acceptable than “*A thief chases an officer*”). To minimize the effect of general memory demands, a whole sentence of a minimal length was visually presented for a longer time than was needed to respond.

In Japanese syntax, the grammatical relations are first marked by grammatical particles (nominative, dative, or accusative), which in turn allow the assignment of semantic roles. In the active sentences we used, a noun phrase with the nominative case marker -*ga* (*green* letters in Figure 6B) is associated with an agent, and the one with the accusative case marker -*o* is associated with a patient. For the passive sentences we used, however, a noun phrase with the nominative case marker -*ga* is associated with an experiencer (a person experiencing a situation), whereas a passive bound verb “-(*r*)*areru*” marks passiveness, making a subject-verb pair with the experiencer. In contrast, a noun phrase with the dative marker -*ni* is associated with an agent, whereas an action verb (e.g., “*hik*(*u*),” “*pull*”) makes a subject-verb pair with the agent, forming a subordinate clause within the main clause “°-*ga.*... -(*r*)*areru.*” Note that there exist similar causative structures in both Japanese and English: “*Hanako-ga kare-ni hik-aseta*,” “*Hanako made him pull*.” Actually, there are two types of passivization in Japanese: *ni* passive (e.g., “*Hanako-ga Taro-ni hik-areru*,” “*Hanako is affected by Taro's pulling her*”) and *ni yotte* passive (e.g., “*Hanako-ga Taro-ni yotte hik-areru*,” “*Hanako is pulled by Taro*”). According to Kuroda (1992), the *ni* passive involves no noun-phrase movement, while the *ni yotte* passive involves a movement similar to the case in English. For the scrambled sentences, an object moves from its canonical position to higher nodes by undergoing another Merge operation. This type of constructions is perfectly normal, not only in Japanese but in German, Finnish, and other languages. We also tested the One-argument condition, under which each sentence was presented with an intransitive verb and double agents. This condition did not involve two-argument relationships, and was thus syntactically simpler than any of the Two-argument conditions.

### Hypothesis III

Here we present the following hypothesis (Hypothesis III):

(3) The DoM domain changes dynamically in accordance with iterative Merge applications, the Search distances, and/or task requirements.

Since Merge combines two syntactic objects to form a larger structure, Merge always produces a one-level higher node. When Merge applies iteratively to an existing phrase or sentence, the DoM domain becomes thus larger in accordance with the number of Merge applications. The Search distance is the structural distance between two distinct parts to which the Search operation applies, regardless of the nodes that are irrelevant to the Search operation. As observed from Figure 4, the DoM domain changes in accordance with the Search distance. On the other hand, for every sentence stimulus in the study of Ohta et al. (2013), the construction of syntactic structures was ensured by task requirements, in which three sentence types had to be distinguished while they were completely mixed. Task requirements include not only certain constraints required by experimental tasks, but detailed parsing naturally required to understand a part of phrases or sentences (e.g., subject-verb relationships and noun-pronoun (coreference) relationships).

In the above mentioned paradigm (Kinno et al., 2008), the four task conditions (three sentence types under the Two-argument conditions, as well as one type under the One-argument condition) were completely mixed (see Figure 6A). With such task requirements, the DoM domain spanned three relevant words for all sentence types under the Two-argument conditions. Under the One-argument condition, the action of two stick figures was always identical, and thus a subject (a triangle just below N in Figure 6B) is regarded as a unit. Under these four task conditions, participants were required to check at least one of the argument-verb relationships, demanding Search at least once. For the scrambled sentences alone, an additional Search operation should match the identical indices of the moved object and its trace. For the active, passive, and scrambled sentences, the estimates of DoM were 2, 3, and 3, respectively, while those of the DoM was 1 under the One-argument condition.

### Applying the doM to various sentence types

**Figure 7:**
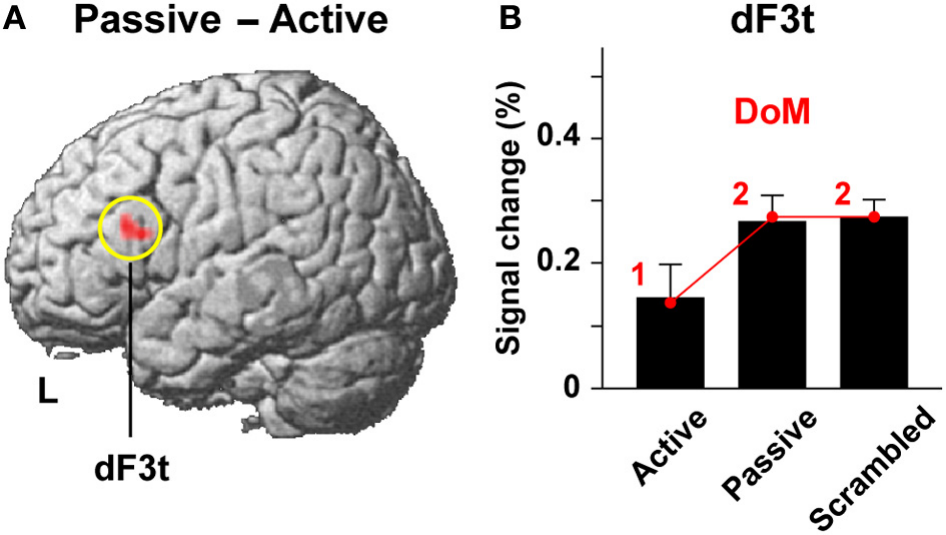
**Activations in the L. dF3t modulated by the DoM. (A)** A region identified by the Passive – Active contrast (see Figure 6). Activations were projected onto the left (L) lateral surface of a standard brain. **(B)** Percent signal changes for the active, passive, and scrambled sentence conditions in the L. dF3t, taking the One-argument condition as a reference. Overlaid red dots and lines denote the values fitted with the estimates (digits in red) for the model of the DoM.

In the study of Kinno et al. (2008), we directly contrasted passive and active sentence conditions to identify a cortical region that is activated by purely syntactic processes. This stringent contrast resulted in significant activation in the left dorsal F3t (L. dF3t) alone [(−48, 24, 21), *Z* = 3.8] (Figure 7A), which was very close to the L. F3op/F3t activation in the study of Ohta et al. (2013). The L. dF3t activation was significantly enhanced under both the passive and scrambled sentence conditions compared to that under the active sentence condition (*P* ≤ 0.033) (Figure 7B), whereas there was no significant difference between the passive and scrambled sentence conditions (*P* = 0.15). Taking the One-argument condition as a reference for subtracted estimates, the signal changes in the L. dF3t were precisely correlated in a step-wise manner with the parametric model of the DoM [1, 2, 2], producing the RSS of 0.0001 and *r*^2^ of 0.99, without significant deviation for the three contrasts (*P* ≥ 0.87). The model of the DoM thus *sufficiently* explains the L. dF3t activations. It should be noted that the parametric model of “the number of nodes” [2, 4, 4] also yielded the same fitting results in this case. The design of experimental paradigms limits the separation of multiple factors.

In a recent fMRI study, only right-branching constructions were examined, and activations in the L. F3t were modulated by the size of constituents (i.e., number of terminal nodes) (Pallier et al., 2011). Since the estimates of the DoM were identical to those of “the number of Merge” or “the number of non-terminal nodes” in this case, it was not possible to separate these factors. Taking their simplest condition (lists of unrelated words) as an appropriate reference, the model of the DoM actually showed a comparable or better goodness of fit for activations in the L. F3t, when compared with their log-fitting models.

## Further confirmation of hypothesis III

### The effect of the search distances on the DoM

**Figure 8:**
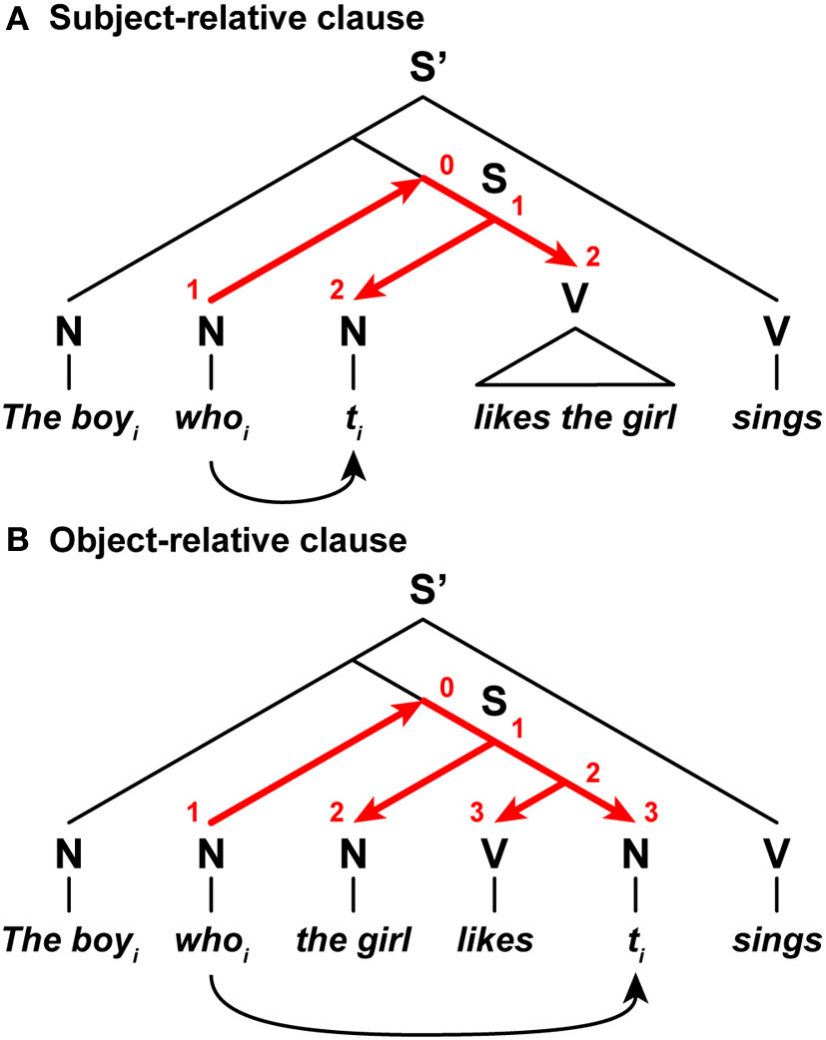
**The DoM domains varied with the Search distances. (A)** A sentence with a subject-relative clause. **(B)** A sentence with an object-relative clause. In these relative clauses, a relative pronoun *who_i_* is displaced from its subject or object position denoted by a trace *t_i_*. A set of red straight arrows corresponds to the DoM domain. The digits shown in red denote the DoM for each node within the domain. Symbols used: S and S', sentence; N, noun phrase; V, verb phrase; *t_i_*, trace (subscripts denote the same entity).

Neuroimaging and psycholinguistic studies have reported that English sentences with object-relative clauses have higher processing loads than those with subject-relative clauses (Just et al., 1996; Stromswold et al., 1996; Gibson, 2000). To properly parse the relative clauses, the relative pronoun and its antecedent are coindexed; “*who_i_*” and “*the boy_i_*,” respectively, in the example shown in Figure 8. In a subject-relative clause, a relative pronoun “*who_i_*” was displaced from the *subject* position denoted by a trace *t_i_* (originally, “*the boy_*i*_ likes the girl*”), while in an object-relative clause, a relative pronoun was displaced from the *object* position (originally, “*the girl likes the boy_i_*”). Following the proposal by Hawkins (1999), we assume that the relative pronoun searches the corresponding trace within tree structures of a sentence (see curved arrows in Figure 8). In a subject-relative clause, Search ends at the initiation of the verb phrase, while in an object-relative clause, Search ends *after* a verb appears within a subordinate clause. In accordance with the Search distances for these examples, the DoM would become one unit larger for the object-relative clause than the subject-relative one. Higher processing loads observed with object-relative clauses are consistent with this inference about the DoM domain.

### The effect of task requirements on the DoM

If Hypothesis III is correct, then the L.F3op/F3t activations can be different in accordance with task requirements, even when the same sentences are presented. In our previous fMRI study, we compared three explicit linguistic tasks with the same set of normal two-word sentences: syntactic decision, semantic decision, and phonological decision tasks (Suzuki and Sakai, 2003). In the syntactic decision task, the participants judged whether or not the presented sentence was syntactically correct, and this judgment required syntactic knowledge about the distinction between transitive and intransitive verbs (e.g., normal sentence, “*yuki-ga tumoru*,” “*snow lies* (*on the ground*)”; anomalous sentence, “*yuki-o tumoru*,” “(*something*) *lies snow*”). In the semantic decision task, lexico-semantic knowledge about selectional restrictions was indispensable. In the phonological decision task, phonological knowledge about accent patterns was required. Neither the semantic decision task nor the phonological decision task, both with *implicit* syntactic processing, elicited significant activations in the L. F3op/F3t (−57, 9, 6), which was significantly activated during *explicit* syntactic processing, even by a direct comparison between the syntactic decision task and the other tasks. These results suggest the presence of the DoM domain in accordance with the task requirements of explicit syntactic processing.

### The mixed effects of the search distances and task requirements on the DoM

**Figure 9:**
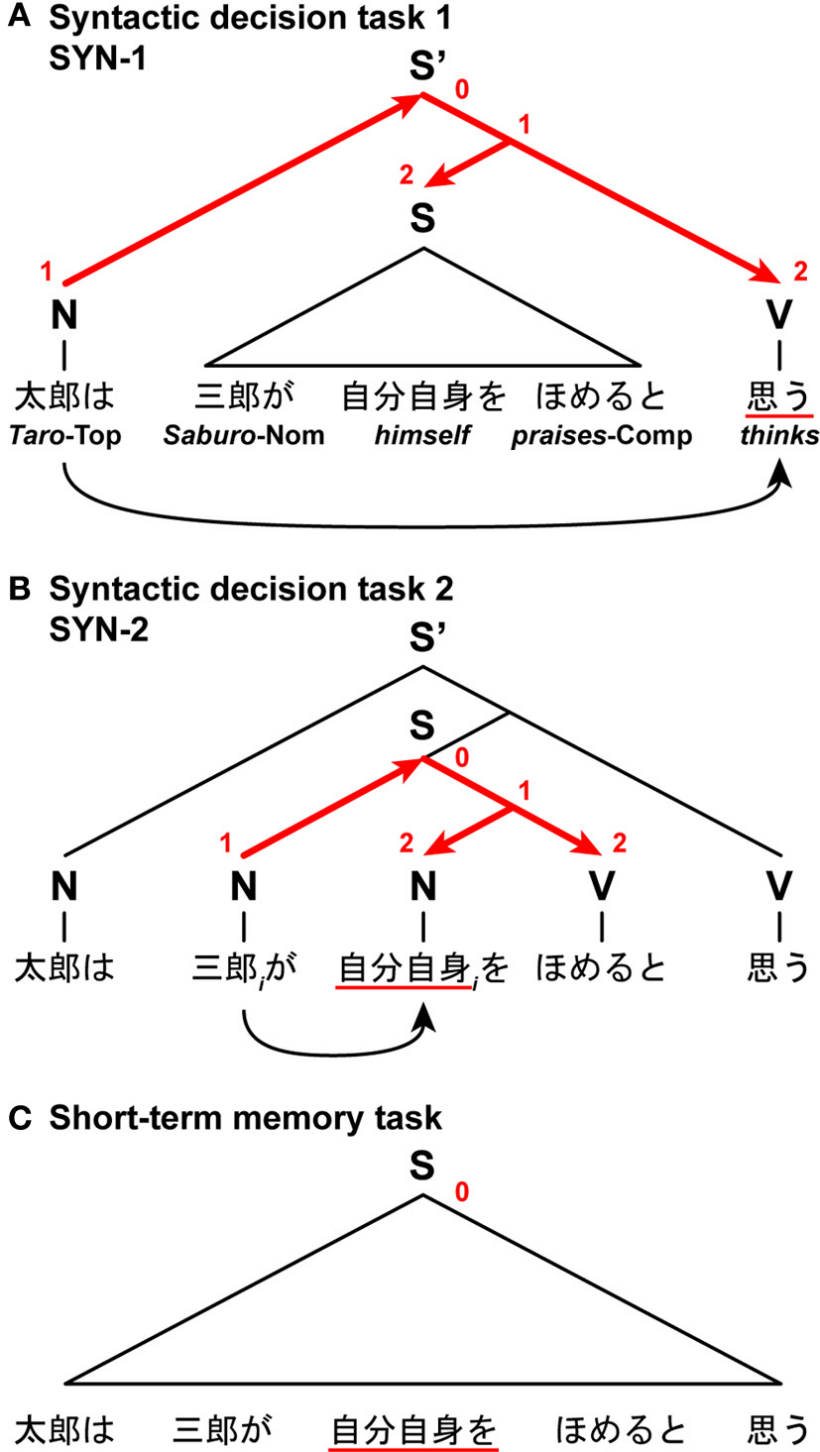
**The DoM domains varied with the Search distances and task requirements.** In this task, participants read Japanese nested sentences (“*Taro-wa Saburo-ga jibunjishin-o homeru-to omou*,” “*Taro thinks that Saburo praises himself*”), in which phrases were sequentially presented. **(A)** A syntactic decision task 1, in which participants judged subject-verb relationships. A set of red straight arrows corresponds to the DoM domain. The digits shown in red denote the DoM for each node within the domain. **(B)** A syntactic decision task 2, in which participants judged noun-pronoun (coreference) relationships (subscripts denote the same entity). **(C)** A short-term memory task with a sentence, in which participants judged the temporal order of the phrases. Symbols used: S and S', sentence; N, noun phrase; V, verb phrase; Top, topic; Nom, nominative case; Comp, complementizer.

In another fMRI study, we directly compared syntactic decision and short-term memory tasks (Hashimoto and Sakai, 2002). In this unique paradigm, we visually presented nested sentences that included two proper nouns, two verbs, and one pronoun, in which either verb or pronoun was underlined. After presenting one complete sentence in a phrase-by-phrase manner, paired phrases including an underlined phrase were shown. In one syntactic decision task (SYN-1), participants were required to judge whether the subject of an underlined verb corresponded to the person in paired phrases (Figure 9A). In this case, the Search distance was the structural distance between the subject and verb of the same clause. In the other syntactic decision task (SYN-2), the participants were required to judge whether an underlined pronoun was able to refer to the person in paired phrases (Figure 9B). In this case, the Search distance was the structural distance between the coindexed noun and pronoun. In these syntactic decision tasks, the Search distance, and consequently the DoM domain, changed dynamically in accordance with the different task requirements, even when the same sentences were presented. The estimate of the resultant DoM was 2 for both cases. In a short-term memory task with a sentence, the participants memorized the linear order of the phrases, and judged whether the left-hand phrase preceded the right-hand one in the original sequence (Figure 9C). With such a task requirement, the factor of DoM would become less effective. Indeed, we found that activations in the L. F3op/F3t were equally enhanced in both syntactic decision tasks when compared with the short-term memory task.

## Conclusions

In this article, we reviewed recent advances in theoretical linguistics and functional neuroimaging in the following respects. First, we provided theoretical discussions about the hierarchical tree structures of sentences, and introduced the two fundamental linguistic operations of Merge and Search. We also presented our hypotheses that the DoM is a key computational concept to properly measure the complexity of tree structures (Hypothesis I), and that the basic frame of the syntactic structure of a given linguistic expression is determined essentially by functional elements, which trigger Merge and Search operations (Hypothesis II). Second, we presented our recent fMRI studies, which have demonstrated that the DoM, together with the number of Search, is indeed a key syntactic factor that accounts for syntax-selective activations in the L. F3op/F3t and L. SMG (Ohta et al., 2013). Moreover, based on the DCM and DTI results, we revealed the significance of the top-down connection from the L. F3op/F3t to L. SMG, suggesting that information about the DoM is transmitted through this specific dorsal pathway. Third, we further hypothesized that the DoM domain changes dynamically in accordance with iterative Merge applications, the Search distances, and/or task requirements (Hypothesis III). We showed that the DoM sufficiently explains activation modulations due to different structures reported in previous fMRI studies (Kinno et al., 2008; Pallier et al., 2011). Finally, we confirmed that Hypothesis III accounts for higher processing loads observed with object-relative clauses, as well as activations in the L. F3op/F3t during explicit syntactic decision tasks, reported in the previous neuroimaging and psycholinguistic studies (Just et al., 1996; Stromswold et al., 1996; Gibson, 2000; Hashimoto and Sakai, 2002; Suzuki and Sakai, 2003). It is likely that the DoM serves as a key computational principle for other human-specific cognitive capacities, such as mathematics and music, both of which can be expressed by hierarchical tree structures. A future investigation into the computational principles of syntax will further deepen our understanding of uniquely human mental faculties.

### Conflict of interest statement

The authors declare that the research was conducted in the absence of any commercial or financial relationships that could be construed as a potential conflict of interest.
